# Bionic Energy-Efficient Inverse Kinematics Method Based on Neural Networks for the Legs of Hydraulic Legged Robots

**DOI:** 10.3390/biomimetics10060403

**Published:** 2025-06-14

**Authors:** Jinbo She, Xiang Feng, Bao Xu, Linyang Chen, Yuan Wang, Ning Liu, Wenpeng Zou, Guoliang Ma, Bin Yu, Kaixian Ba

**Affiliations:** 1 School of Mechanical Engineering, Yanshan University, Qinhuangdao 066004, China; sjb@stumail.ysu.edu.cn (J.S.); fengxiang@stumail.ysu.edu.cn (X.F.); xubao@stumail.ysu.edu.cn (B.X.); cly@stumail.ysu.edu.cn (L.C.); wy1996@stumail.ysu.edu.cn (Y.W.); liuning23@stumail.ysu.edu.cn (N.L.); xrl@stumail.ysu.edu.cn (W.Z.); yb@ysu.edu.cn (B.Y.); 2 The State Key Laboratory of Crane Technology, Yanshan University, Qinhuangdao 066004, China

**Keywords:** hydraulic legged robot, redundant degree of freedom (RDOF), energy-saving, inverse kinematics, neural network (NN)

## Abstract

Hydraulic legged robots, with advantages such as high load capacity and power density, have become a strategic driving force in advancing intelligent mobile platform technologies. However, their high energy consumption significantly limits long-duration endurance and efficient operational performance. In this paper, inspired by the excellent autonomous energy-efficient consciousness of mammals endowed by natural evolution, a bionic energy-efficient inverse kinematics method based on neural networks (EIKNN) is proposed for the energy-efficient motion planning of hydraulic legged robots with redundant degrees of freedom (RDOFs). Firstly, the dynamic programming (DP) algorithm is used to solve the optimal joint configuration with minimum energy loss as the goal, and the training data set is generated. Subsequently, the inverse kinematic model of the leg with minimum energy loss is learned based on neural network (NN) simulation of the autonomous energy-efficient consciousness endowed to mammals by natural evolution. Finally, extensive comparative experiments validate the effectiveness and superiority of the proposed method. This method not only significantly reduces energy dissipation in hydraulic legged robots but also lays a crucial foundation for advancing hydraulic legged robot technology toward high efficiency, environmental sustainability, and long-term developmental viability.

## 1. Introduction

Robotics, as a key technology carrier of intelligent and autonomous development in the future, has become an important research direction in many fields in recent years [1,2,3,4]. In recent years, the development of tactile perception and human–computer interactions has further promoted the application of robots [5,6,7]. Legged robots, with their unique mammal-like leg locomotion mechanism, have demonstrated excellent unstructured environmental adaptability and flexible locomotion characteristics [8,9,10]. Among them, mammals, as a typical hydraulic-driven system [11], endow the bionic hydraulic legged robot with the advantage of higher power density, which makes it outstanding in terms of carrying capacity and dynamic stability [12,13,14]. In addition, the series configuration designed with redundant degrees of freedom (RDOFs) further enhances the workspace and locomotion flexibility of the hydraulic legged robot [15,16,17,18]. However, the inherent energy loss problem of the hydraulic legged robots significantly constrains the robot’s continuous operation ability in the field environment [19,20]. Fortunately, RDOFs endow the inverse kinematics of the hydraulic legged robot with multiple solutions [21], which provides a potential solution space for optimizing the energy loss of the system. Furthermore, in nature, many organisms are constantly evolving to adapt to the changing demands of the environment [11]. And mammals are endowed with an excellent sense of autonomous energy-efficient conservation through natural evolution and learning over time, and can fulfill the task requirements with lower energy loss [22]. Therefore, based on the RDOF characteristics of the hydraulic legged robot and the autonomous energy conservation awareness of mammals, it is an important technical prerequisite to realize the long-term endurance and efficient operation of the hydraulic legged robot by using the neural network (NN) to learn the inverse kinematics model with the minimum energy loss and reduce the energy loss during the movement of the hydraulic legged robot.

It is worth noting that the introduction of RDOFs not only increases the difficulty of controlling the robot but also significantly increases the difficulty of solving inverse kinematics [23]. In order to address the effect of RDOFs on the control performance of a robot, Ba et al. solve an inverse kinematics model of a serial robot with RDOFs using three three-point collinear constraints. And based on this redundancy characteristic, a virtual-constraints-based end-effector pose compensator (VEPC) method is proposed, which significantly improves the end pose control accuracy of a redundant serial robot [24]. However, when the VEPC method is oriented to multiple RDOFs, the difficulty of solving will increase exponentially. Fortunately, with its excellent feature learning and nonlinear mapping capabilities, NN has been widely used in model construction and has become a core technology for efficient and accurate modeling in many fields [25,26,27,28,29,30]. Li et al. established an inverse kinematics model for a robot with RDOFs using the NN model and achieved a good control effect [31,32]. In addition, in recent years, researchers have given a variety of solutions to the inverse kinematics problem of RDOFs from different angles. Guo et al. explore a pseudo-inverse-based PID path planning method for a robot with RDOFs in noisy environments [33]. Boudreau et al. solve the inverse kinematics model of a redundant robot using the optimization approach [34]. Although the above methods have carried out an in-depth analysis on the inverse kinematics problem of RDOFs, the energy loss problem of the robot has not been studied in depth.

To address this problem, researchers have deeply explored the influence of robot trajectory planning on its energy loss from a bionic perspective. Hoyt et al. explored the effect of gait parameters on the energy loss of a horse and tracked the overall energy loss of the horse in real time by monitoring the oxygen consumption of horses [35]. Subsequently, the team conducted a detailed study on multiple gait parameters such as stride frequency and the time proportion of the contact phase and swing phase [36]. Kun et al. used the pattern search method to optimize the common foot-end trajectory of the quadruped robot, such as the cubic curve and the cosine cycloid, to further reduce the energy consumption of the robot [37]. In addition, in order to save energy from the power source of the robot, Cui et al. proposed a control method to reduce the energy consumption of the robot. The pressure of the system is controlled by load sensing, which reduces the power consumption of the system by more than 50% [38]. Although the above studies are related to energy-saving from the robot’s trajectory planning and hydraulic power source, the mapping relationship between RDOFs and energy-saving inverse kinematics solution sets has not yet been established, resulting in the robot’s inability to achieve global energy optimization through redundancy characteristics. Therefore, for the RDOFs of the hydraulic legged robot, how to use an NN to learn the energy-efficient inverse kinematics model with minimum energy loss is still an urgent problem to be solved.

In view of the above problems, a bionic energy-efficient inverse kinematics method based on neural networks (EIKNN) for the legs of hydraulic legged robots is innovatively proposed in this paper. Specifically, the single leg of the hydraulic legged robot with RDOFs is taken as the research object. Firstly, the spatial mapping relationship between the hydraulic drive unit (HDU) and the joint angle is derived, and the foot-end motion space under the joint motion limit is analyzed. The inverse kinematics model of the single leg of the hydraulic legged robot is established. Then, an energy loss model for evaluating leg motion is established by considering the along-travel loss of the oil and the friction loss of the HDU motion, and the dynamic programming (DP) algorithm is used to solve the optimal joint configuration based on the principle of minimum energy loss during motion. Finally, taking the random foot-end trajectory and the corresponding optimal joint configuration as the training samples, the NN is used to learn the energy-efficient inverse kinematics model of the single leg of the hydraulic legged robot. The innovation of this paper is as follows:

(1) A novel EIKNN method is proposed. Based on the multi-solution characteristics of the inverse kinematics of the leg of the hydraulic legged robot with RDOFs, an NN is used to learn the inverse kinematics model under the minimum energy loss by referring to the autonomous energy-efficient consciousness of mammals.
(2) An energy loss model for the leg motion of the hydraulic legged robot with RDOFs is proposed. An energy loss function for evaluating the leg motion is designed based on the along-travel loss of the oil and the friction loss of the HDU motion.
(3) This method not only ensures effective motion accuracy but also has an excellent energy-saving effect, which can effectively improve the endurance of the hydraulic legged robot in the field and reduce the application cost of the hydraulic legged robot.

## 2. Mathematical Modeling of the Leg of Hydraulic Legged Robot with RDOFs

### 2.1. Mapping of the Drive Space

**Figure 1:**
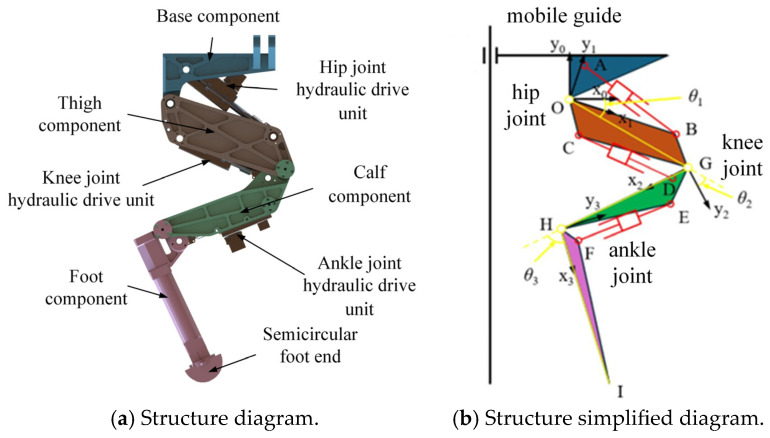
Structure of 3-DOF hydraulic leg of hydraulic legged robot.

The 3-degree-of-freedom (DOF) hydraulic single leg is a typical serial mechanism with RDOFs. In this study, the 3-DOF hydraulic single leg of a hydraulic legged robot shown in Figure 1a is taken as the research object. It is worth noting that different from the typical joint rotation to drive the leg movement, the hip joint, knee joint, and ankle joint of the 3-DOF hydraulic single leg are each driven by an HDU to drive the joint rotation, so that the foot-end motion trajectory is determined by the displacement of the HDU piston rod of each joint. Therefore, the drive space of the HDU needs to be mapped to the joint motion space.

**Figure 2:**
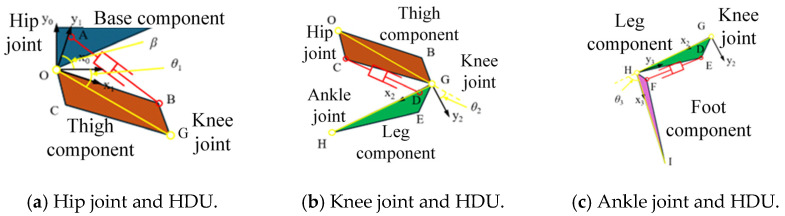
The schematic diagram of each joint and the corresponding HDU position.

The dimensions and relative positions of the leg components are shown in Figure 1b. In order to realize the mapping from the HDU drive space to the joint motion space, the positional relationship between each joint and the corresponding HDU is separated as shown in Figure 2. And the geometric method and the geometric parameters of the leg components in Table 1 are further utilized to solve the geometric relationship between the displacement of the joint HDU piston rod and the corresponding joint angle. Since the specific derivation process is not the core of this paper, the final expression for the drive space transformation will be given directly in this paper.

The expression between the angle of each joint of the 3-DOF hydraulic single leg and the displacement of the corresponding HDU piston rod is as follows:

(1) $$\left\{\begin{array}{l} \theta_{1}=\beta -\arccos\left(\frac{OA^{2}+OB^{2}-{\left(l_{01}+\Delta x_{p1}\right)}^{2}}{2\cdot OA\cdot OB}\right)-∠BOG\\ \theta_{2}=\arccos\left(\frac{GC^{2}+GD^{2}-{\left(l_{02}+\Delta x_{p2}\right)}^{2}}{2\cdot GC\cdot GD}\right)-\pi -∠HGD+∠OGC\\ \theta_{3}=\pi -\arccos\left(\frac{EH^{2}+HF^{2}-{\left(l_{03}+\Delta x_{p3}\right)}^{2}}{2\cdot EH\cdot HF}\right)-∠IHF-∠GHE \end{array}\right.$$

where $\theta_{1}$ is the rotation angle of the hip joint, $\theta_{2}$ is the rotation angle of the knee joint, $\theta_{3}$ is the rotation angle of the ankle joint, $l_{01}$ is the initial elongation of the HDU piston rod of the hip joint, $l_{02}$ is the initial elongation of the HDU piston rod of the knee joint, $l_{03}$ is the initial elongation of the HDU piston rod of the ankle joint, $\Delta x_{p1}$ is the change in displacement of the HDU piston rod of the hip joint, $\Delta x_{p2}$ is the change in displacement of the HDU piston rod of the knee joint, and $\Delta x_{p3}$ is the change in displacement of the HDU piston rod of the ankle joint. The other parameters are structural parameters.

Based on Equation (1), the expression for the change in displacement of the HDU piston rod at each joint with respect to the joint angle can be obtained as

(2) $$\left\{\begin{array}{l} \Delta x_{p1}=\sqrt{OA^{2}+OB^{2}-2\cdot OA\cdot OB\cdot \cos\left(\beta -∠BOG-\theta_{1}\right)}-l_{01}\\ \Delta x_{p2}=\sqrt{GC^{2}+GD^{2}-2\cdot GC\cdot GD\cdot \cos\left(∠OGC-\theta_{2}-\pi -∠HGD\right)}-l_{02}\\ \Delta x_{p3}=\sqrt{EH^{2}+HF^{2}-2\cdot EH\cdot HF\cdot \cos\left(\theta_{3}-\pi +∠IHF+∠GHE\right)}-l_{03} \end{array}\right.$$

In order to facilitate the subsequent calculation of the energy loss of 3-DOF hydraulic single-leg motion, the mapping relationships between the motion velocity and acceleration of the HDU piston rod and the joint angle, angular velocity, and angular acceleration are further derived.

For the mapping in velocity space, first-order differentiation of Equation (2) with respect to time t yields an expression for the velocity of the HDU piston rod with respect to the joint angle and angular velocity as

(3) $$\left\{\begin{array}{l} {\dot{x}}_{p_{1}}=\frac{OA\cdot OB\cdot \sin(∠BOG-\beta +\theta_{1})\cdot {\dot{\theta }}_{1}}{\sqrt{\left(OA^{2}-2\cdot \cos(∠BOG+\theta_{1}-\beta )\cdot OA\cdot OB+OB^{2}\right)}}\\ {\dot{x}}_{p_{2}}=\frac{-(GC\cdot GD\cdot \sin(∠HGD-∠OGC+\theta_{2})\cdot {\dot{\theta }}_{2}}{\sqrt{\left(GC^{2}+2\cdot \cos(∠HGD-∠OGC+\theta_{2})\cdot GC\cdot GD+GD^{2}\right)}}\\ {\dot{x}}_{p_{3}}=\frac{-(EH\cdot HF\cdot \sin(∠IHF+∠GHE+\theta_{3})\cdot {\dot{\theta }}_{3}}{\sqrt{\left(EH^{2}+2\cdot \cos(∠IHF+∠GHE+\theta_{3})\cdot EH\cdot HF+HF^{2}\right)}} \end{array}\right.$$

For the mapping in acceleration space, first-order differentiation of Equation (3) with respect to time t yields the following expressions for the HDU piston rod acceleration with respect to joint angle, angular velocity, and angular acceleration as

(4) $$\left\{\begin{array}{cc} {\ddot{x}}_{p_{1}} & ={\dot{\theta }}_{1}^{2}\cdot (\frac{\left(OA\cdot OB\cdot \cos(∠BOG-\beta +\theta_{1})\right)}{\sqrt{\left(OA^{2}-2\cdot \cos(∠BOG+\theta_{1}-\beta )\cdot OA\cdot OB+OB^{2}\right)}}-\\ & \frac{\left(OA^{2}\cdot OB^{2}\cdot \sin{\left(∠BOG-\beta +\theta_{1}\right)}^{2}\right)}{{\left(OA^{2}-2\cdot \cos(∠BOG+\theta_{1}-\beta )\cdot OA\cdot OB+OB^{2}\right)}^{\left(3/2\right)}})+\\ & \frac{\left(OA\cdot OB\cdot {\ddot{\theta }}_{1}\cdot \sin(∠BOG-\beta +\theta_{1})\right)}{\sqrt{\left(OA^{2}-2\cdot \cos(∠BOG+\theta_{1}-\beta )\cdot OA\cdot OB+OB^{2}\right)}}\\ {\ddot{x}}_{p_{2}} & =-{\dot{\theta }}_{2}^{2}\cdot (\frac{\left(GC\cdot GD\cdot \cos(∠HGD-∠OGC+\theta_{2})\right)}{\sqrt{\left(GC^{2}+2\cdot \cos(∠HGD-∠OGC+\theta_{2})\cdot GC\cdot GD+GD^{2}\right)}}+\\ & \frac{\left(GC^{2}\cdot GD^{2}\cdot \sin{\left(∠HGD-∠OGC+\theta_{2}\right)}^{2}\right)}{{\left(GC^{2}+2\cdot \cos(∠HGD-∠OGC+\theta_{2})\cdot GC\cdot GD+GD^{2}\right)}^{\left(3/2\right)}})-\\ & \frac{\left(GC\cdot GD\cdot {\ddot{\theta }}_{2}\cdot \sin(∠HGD-∠OGC+\theta_{2})\right)}{\sqrt{\left(GC^{2}+2\cdot \cos(∠HGD-∠OGC+\theta_{2})\cdot GC\cdot GD+GD^{2}\right)}}\\ {\ddot{x}}_{p_{3}} & =-{\dot{\theta }}_{3}^{2}\cdot (\frac{\left(EH\cdot HF\cdot \cos(∠IHF+∠GHE+\theta_{3})\right)}{\sqrt{\left(EH^{2}+2\cdot \cos(∠IHF+∠GHE+\theta_{3})\cdot EH\cdot HF+HF^{2}\right)}}+\\ & \frac{\left(EH^{2}\cdot HF^{2}\cdot \sin{\left(∠IHF+∠GHE+\theta_{3}\right)}^{2}\right)}{{\left(EH^{2}+2\cdot \cos(∠IHF+∠GHE+\theta_{3})\cdot EH\cdot HF+HF^{2}\right)}^{\left(3/2\right)}})-\\ & \frac{\left(GF\cdot HF\cdot {\ddot{\theta }}_{3}\cdot \sin(∠IHF+∠GHE+\theta_{3})\right)}{\sqrt{\left(GF^{2}+2\cdot \cos(∠IHF+∠GHE+\theta_{3})\cdot GF\cdot HF+HF^{2}\right)}} \end{array}\right.$$

### 2.2. Analysis of the Foot-End Movement Space

Once the mapping relationship between the HDU piston rod displacement and joint angle is obtained, the foot-end motion space needs to be determined to provide boundaries for subsequent NN input samples. By determining the HDU physical limits of each joint, the rotation limits of each joint are obtained using the drive space mapping relations in Section 2.1, and the Jacobi matrix is used to obtain the final foot-end motion limits. Therefore, it is necessary to obtain HDU physical limits and the rotation limits of each joint. The physical limits of HDU and the rotation limits of each joint obtained in this study, using methods such as scientific measurements and valid estimation, are shown in Table 2.

**Figure 3:**
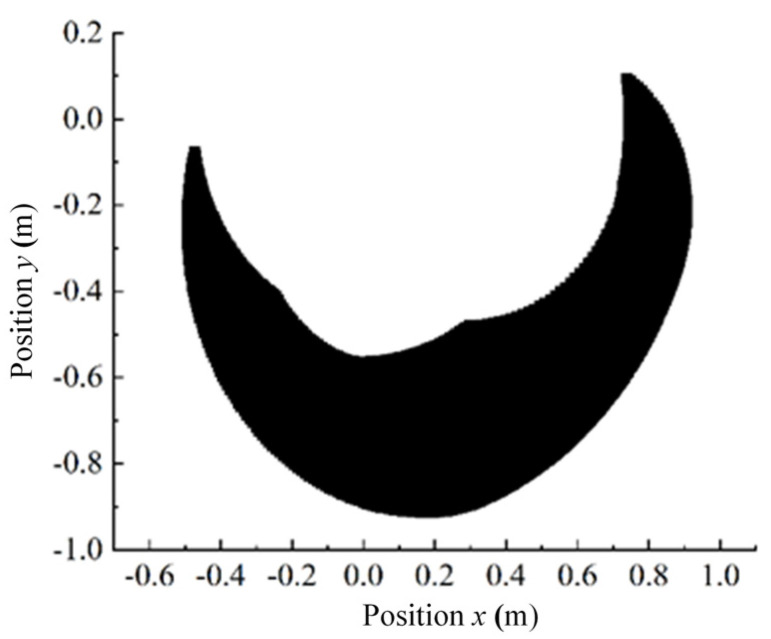
Foot motion space of 3-DOF hydraulic single leg.

Secondly, the obtained physical limits of the HDU and the rotation limits of each joint are used to derive the foot-end limits to analyze the foot-end motion space. As shown in Figure 1b, the hip joint coordinate system ($x_{0}$, $y_{0}$) is established, whose origin O is the location of the hip joint, and the forward kinematic expression for the 3-DOF hydraulic single leg is obtained as

(5) $$\left\{\begin{array}{c} x=l_{1}\cos\left(\theta_{1}\right)+l_{2}\cos\left(\theta_{1}+\theta_{2}\right)+l_{3}\cos\left(\theta_{1}+\theta_{2}+\theta_{3}\right)\\ y=l_{1}\sin\left(\theta_{1}\right)+l_{2}\sin\left(\theta_{1}+\theta_{2}\right)+l_{3}\sin\left(\theta_{1}+\theta_{2}+\theta_{3}\right) \end{array}\right.$$

where x is the position of the foot end on the x axis in the hip coordinate system, y is the position of the foot end on the y axis in the hip coordinate system, $l_{1}$ is the length of the thigh component, corresponding to the component OG, $l_{2}$ is the length of the calf component, corresponding to the component GH, and $l_{3}$ is the length of the foot-end component, corresponding to the component HI. According to Equation (5) and Table 2, the foot-end motion space of a 3-DOF hydraulic single leg can be obtained as shown in Figure 3. In addition, both the subsequent training samples and the motion control need to strictly ensure that the foot-end position is within that motion space.

Define the position of the foot end as $X={\left[\begin{array}{cc} x & y \end{array}\right]}^{T}$, the velocity of the foot end as $\dot{X}={\left[\begin{array}{cc} \dot{x} & \dot{y} \end{array}\right]}^{T}$, the acceleration of the foot end as $\ddot{X}={\left[\begin{array}{cc} \ddot{x} & \ddot{y} \end{array}\right]}^{T}$, the joint angle as $\theta ={\left[\begin{array}{ccc} \theta_{1} & \theta_{2} & \theta_{3} \end{array}\right]}^{T}$, the angular velocity of the joint as $\dot{\theta }={\left[\begin{array}{ccc} {\dot{\theta }}_{1} & {\dot{\theta }}_{2} & {\dot{\theta }}_{3} \end{array}\right]}^{T}$, the angular acceleration of the joint as $\ddot{\theta }={\left[\begin{array}{ccc} {\ddot{\theta }}_{1} & {\ddot{\theta }}_{2} & {\ddot{\theta }}_{3} \end{array}\right]}^{T}$, and the Jacobi matrix as J(θ). Then, the foot-end position is first-order differentiated with respect to time t to obtain the following expression for the velocity of the foot-end motion of the 3-DOF hydraulic single leg with respect to the angles and angular velocities of each joint as

(6) $$\dot{X}=J\left(\theta \right)\dot{\theta }=J_{1}{\dot{\theta }}_{1}+J_{2}{\dot{\theta }}_{2}+J_{3}{\dot{\theta }}_{3}$$

Equation (6) is again first-order differentiated with respect to time t to obtain the following expression for the foot end acceleration of the 3-DOF hydraulic single leg with respect to the angles of the joints, the angular velocities, and the angular accelerations as

(7) $$\ddot{X}=\dot{J}\left(\begin{array}{cc} \theta & \dot{\theta } \end{array}\right)\dot{\theta }+J\left(\theta \right)\ddot{\theta }$$

A first-order differentiation of the Jacobi matrix $J(\theta )={\left[\begin{array}{ccc} J_{1} & J_{2} & J_{3} \end{array}\right]}^{T}$ with respect to time t yields

(8) $$\dot{J}{\left(\begin{array}{cc} \theta & \dot{\theta } \end{array}\right)}_{ji}=\frac{\partial J{\left(\theta \right)}_{ji}}{\partial \theta }\dot{\theta }=\sum_{n=1}^{3}\frac{\partial J{\left(\theta \right)}_{ji}}{\partial \theta_{n}}{\dot{\theta }}_{n}$$

where j=3 is the state space dimension, and i=3 is the number of joints.

### 2.3. Inverse Kinematics Modeling

**Figure 4:**
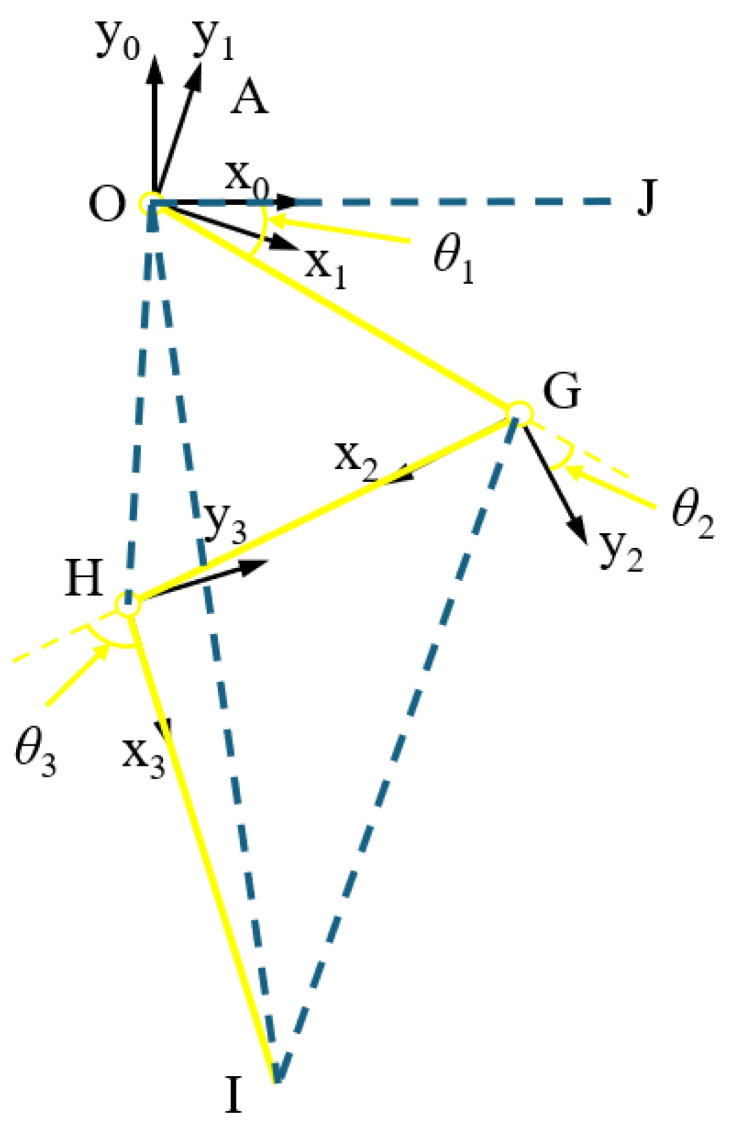
Simplified diagram of 3-DOF hydraulic single-leg connecting rod.

The use of foot-end positions under a random foot-end trajectory to derive the corresponding joint angles requires the creation of the inverse kinematics model based on the 3-DOF hydraulic single leg, which in turn obtains the NN joint angles output under the input of foot-end positions and constitutes the data set. Therefore, we simplify the 3-DOF hydraulic single leg as shown in Figure 4.

(1) When the hip joint angle is taken as a fixed value, $\theta_{1}$ is a known quantity.

According to the known coordinates of the foot end and $\theta_{1}$, we can obtain

(9) $$∠JOI=\arctan2\left(y,x\right)$$

(10) $$∠GOI=∠JOI-\theta_{1}$$

According to the cosine theorem, the ankle angle can be deduced as

(11) $$GI=\sqrt{OG^{2}+OI^{2}-2\cdot OG\cdot OI\cdot \cos\left(∠GOI\right)}$$

(12) $$∠GHI=\arccos\left(\frac{GH^{2}+HI^{2}-EI^{2}}{2\cdot GH\cdot HI}\right)$$

(13) $$\theta_{3}=\pi -∠GHI$$

According to the cosine theorem, we can obtain

(14) $$∠GIH=\arccos\left(\frac{GI^{2}+HI^{2}-GH^{2}}{2\cdot GI\cdot HI}\right)$$

(15) $$∠OIG=\arccos\left(\frac{OI^{2}+GI^{2}-OG^{2}}{2\cdot OI\cdot GI}\right)$$

Combining Equations (14) and (15), we can obtain

(16) ∠OIH=∠HIG−∠OIG

From this, the knee joint angle is

(17) $$OH=\sqrt{OH^{2}+OI^{2}-2\cdot OH\cdot OI\cdot \cos\left(∠OIH\right)}$$

(18) $$∠OGH=\arccos\left(\frac{OG^{2}+GH^{2}-OH^{2}}{2\cdot OG\cdot GH}\right)$$

(19) $$\theta_{2}=\pi -∠OGH$$

(2) When the knee joint angle is used as a fixed value, $\theta_{2}$ is a known quantity.

According to the known coordinates of the foot end and $\theta_{2}$, we can obtain

(20) $$∠OGH=\pi -\left|\theta_{2}\right|$$

Based on Equation (20), we can obtain

(21) $$OH=\sqrt{OH^{2}+OG^{2}-2\cdot OH\cdot OG\cdot \cos\left(∠OGH\right)}$$

According to the cosine theorem, we can obtain

(22) $$∠GOH=\arccos\left(\frac{OG^{2}+OH^{2}-GH^{2}}{2\cdot OG\cdot OH}\right)$$

(23) $$∠OHG=\arccos\left(\frac{OH^{2}+GH^{2}-OG^{2}}{2\cdot OH\cdot GH}\right)$$

(24) $$∠OHI=\arccos\left(\frac{OH^{2}+HI^{2}-OI^{2}}{2\cdot OH\cdot HI}\right)$$

(25) $$∠HOI=\arccos\left(\frac{OH^{2}+HI^{2}-OI^{2}}{2\cdot OH\cdot HI}\right)$$

According to Equations (22) to (25), the ankle and hip joint angles can be obtained as

(26) $$\left\{\begin{array}{l} \theta_{3\_1}=\pi -∠OHI\\ \theta_{3\_2}=-\left(\pi -∠OHI\right) \end{array}\right.$$

(27) $$\left\{\begin{array}{l} \theta_{1\_1}=\left|∠IOJ\right|+∠IOG\\ \theta_{1\_2}=\left|∠IOJ\right|-∠IOG \end{array}\right.$$

(3) When the ankle joint angle is used as a fixed value, $\theta_{3}$ is a known quantity.

According to the known coordinates of the foot end and $\theta_{3}$, we can obtain

(28) $$∠GHI=\pi -\left|\theta_{3}\right|$$

Based on Equation (28), we can obtain

(29) $$GI=\sqrt{GH^{2}+HI^{2}-2GH\cdot HI\cdot \cos\left(∠GHI\right)}$$

because

(30) $$OI=\sqrt{x^{2}+y^{2}}$$

According to the cosine theorem, we can obtain

(31) $$∠OGI=\arccos\left(\frac{OG^{2}+GI^{2}-OI^{2}}{2\cdot OG\cdot GI}\right)$$

(32) $$∠HGI=\arccos\left(\frac{GH^{2}+GI^{2}-HI^{2}}{2\cdot GH\cdot GI}\right)$$

The knee joint angle $\theta_{2}$ can be obtained as

(33) $$\theta_{2}=-\left(\pi -∠OGI+∠HGI\right)$$

According to the cosine theorem, we can obtain:

(34) $$∠GOI=\arccos\left(\frac{OG^{2}+OI^{2}-GI^{2}}{2\cdot OG\cdot OI}\right)$$

The hip joint angle $\theta_{1}$ can be obtained as

(35) $$\theta_{1}=-\left(\left|∠IOJ\right|-∠GOI\right)$$

## 3. Energy-Saving Analysis of the Leg of Hydraulic Legged Robot with RDOFs

Based on the inverse kinematics model in Section 2.3, the energy loss model of the 3-DOF hydraulic single leg during movement is derived, and indicators are established to provide a basis for subsequent NN training. Therefore, in this section, the energy loss model of valve components and pipelines and the HDU energy loss model are established to calculate the energy loss of the 3-DOF hydraulic single-leg motion.

### 3.1. Energy Loss Model of Valve Components and Pipeline Along the Way

In the hydraulic circuit, the hydraulic components that generate pressure drop losses are mainly valves, auxiliary equipment, and pipeline components. The valve energy loss is modeled by the flow and pressure characteristics of the valve as

(36) $$\Delta p_{HHF}={\left(\frac{\rho }{2}\right)}^{\frac{1}{2m}}{\left(\frac{q_{v}}{C_{q}A_{0}}\right)}^{\frac{1}{m}}$$

where ρ is the density of hydraulic oil, m is the throttling index (m=0.5~1), $q_{v}$ is the flow through the valve port, $A_{0}$ is the flow area of the throttle port, and $C_{q}$ is the flow coefficient of the throttle port.

When a fluid passes through a pipe size change area, local pressure loss occurs due to the impacts, local static area, or eddy current caused by changes in fluid velocity and direction. Therefore, we use the classical model to estimate the resistance loss of the fluid as it passes through the local area as

(37) $$\Delta p_{HHJ}=\zeta \frac{\rho q_{P}^{2}}{2A_{P}^{2}}$$

where $q_{P}$ is the oil flow inside the pipeline, $A_{P}$ is the cross-sectional area of the pipeline, and ζ is the local resistance coefficient.

The resistance along the way generated when the fluid flows in the pipeline mainly occurs in the uniform section with the same flow direction. This type of energy loss is uniformly distributed in the uniform flow area in the pipeline, and the magnitude of the energy loss is directly related to the length of the hydraulic pipeline and the square of the oil flow velocity. Therefore, the resistance model along the pipeline can be expressed as

(38) $$\Delta p_{HHY}=\sum \lambda \cdot \frac{L_{P}}{d}\cdot \frac{\rho q_{P}^{2}}{2A_{P}^{2}}$$

where λ is the resistance coefficient along the way, $L_{P}$ is the length of the hydraulic pipeline along the way, and d is the inner diameter of the hydraulic pipeline.

The value of the resistance coefficient along the way is shown in Table 3.

Therefore, the energy loss model of valve components and pipelines can be expressed as

(39) $$P_{HH}^{w}=\Delta p_{HHF}+\Delta p_{HHJ}+\Delta p_{HHY}$$

The above classical mechanical model is used to estimate the resistance loss of hydraulic oil when passing through the local area, and the resistance model of hydraulic oil along the pipeline, covering the local resistance, resistance loss along the way, and throttle loss of the valve port. These models conform to the physical characteristics of the actual hydraulic system and ensure the fidelity of the energy loss model of valve components and pipeline.

### 3.2. Energy Loss Model of Friction Based on HDU Motion

**Figure 5:**
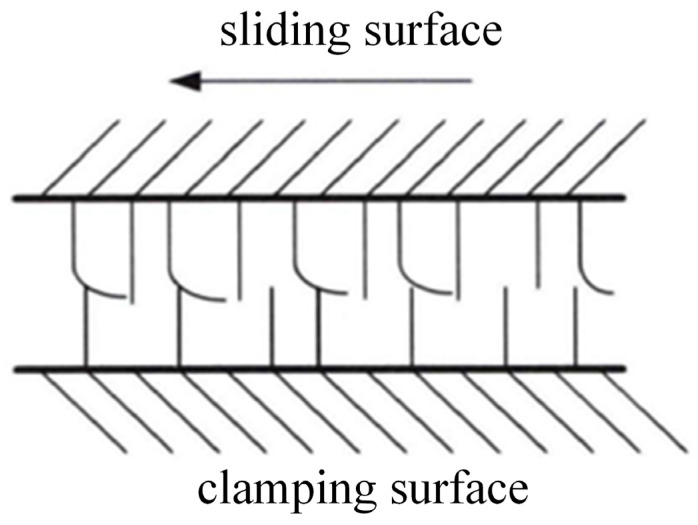
LuGre friction microscopic model.

During 3-DOF hydraulic single-leg motion, the main energy loss from HDU motion is due to friction. Therefore, we use the LuGre friction model to analyze the friction behavior of HDU, the principle of which is shown in Figure 5.

Define z as the state variable describing the average deformation of bristles, specifically:

(40) $$\frac{dz}{dt}=v-\frac{\left|v\right|}{g\left(v\right)}z$$

where v is the sliding surface velocity and $g\left(v\right)>0$ is the Stribeck effect in the friction torque.

(41) $$g\left(v\right)=\frac{1}{\sigma_{0}}[F_{c}+\left(F_{s}-F_{c}\right)e^{-{\left(\frac{v}{v_{s}}\right)}^{2}}]$$

where $F_{c}$ is the Coulomb friction, $F_{s}$ is the maximum static friction, and $v_{s}$ is the Stribeck velocity.

The friction of HDU during motion can be expressed as:

(42) $$F_{f}=\sigma_{0}z+\sigma_{1}\frac{dz}{dt}+\sigma_{2}v$$

where $\sigma_{0}$ is the Rigidity coefficient of bristles, $\sigma_{1}$ is the sliding damping coefficient, and $\sigma_{2}$ is the Viscous friction coefficient. Some of the key parameters are shown in Table 4.

Therefore, the model of energy loss due to friction generated by the motion of HDU can be expressed as

(43) $$P_{HM}^{w}=F_{f}\left|{\dot{x}}_{p}\right|$$

In the friction-based HDU energy loss model, the LuGre friction model can accurately describe the static friction, Coulomb friction, and viscous friction behavior of the hydraulic cylinder. The LuGre model is widely used in industry for high-precision hydraulic control to ensure the accuracy of the friction loss model.

In summary, the 3-DOF hydraulic single leg total energy loss model in this paper can be expressed as

(44) $$E=P_{HH}^{w}+P_{HM}^{w}$$

### 3.3. The Planning of the Foot End and Joint

Based on the inverse kinematics model in Section 2.3, combined with the energy loss model of Equation (44), the NN is trained with the optimization objective of minimum energy loss.

Define $X=[\begin{array}{cc} X & \dot{X} \end{array}]$ as the foot-end state variable and the foot-end state transition equation in the hip coordinate system as

(45) $$X_{k+1}=X_{k}+T_{s}\left(\left[\begin{array}{cc} 0 & 1\\ 0 & 0 \end{array}\right]X_{k}+\left[\begin{array}{c} 0\\ 1 \end{array}\right]{\ddot{x}}_{k}\right)$$

where $X_{k}$ is the foot-end state variable at time k, ${\ddot{x}}_{k}$ is the foot-end acceleration at time k, and $T_{s}$ is the sampling time. During the foot-end motion, different foot-end motion states are obtained by setting different control quantities as a database for subsequent training of the NN, on the premise of satisfying the 3-DOF hydraulic single-leg motion constraint.

In joint planning, based on the parameterized inverse kinematics solution in Section 2.3, such that the hip joint $\theta_{1}$ is a known quantity (the derivation principle is the same when the knee joint $\theta_{2}$ and the ankle joint $\theta_{3}$ are used as known quantities), the state transition equation of the hip joint can be expressed as

(46) $$\theta_{k+1}=\theta_{k}+T_{s}\left(\left[\begin{array}{cc} 0 & 1\\ 0 & 0 \end{array}\right]\theta_{k}+\left[\begin{array}{c} 0\\ 1 \end{array}\right]u_{k}\right)$$

where $\theta_{k}$ is the joint angle state variable at time k, $u_{k}$ is the joint angle control variable at time k, and $u_{k}=\ddot{\theta }$.

According to the above analysis, it can be seen that given a control quantity within the range of inherent joint angular acceleration, the position of the foot end and its corresponding joint angle can be obtained at any point in the foot-end motion space. Combining Equations (6) and (7), the robot joint angular velocity and angular acceleration can be obtained as

(47) $$\left[\begin{array}{c} {\dot{\theta }}_{2}\\ {\dot{\theta }}_{3} \end{array}\right]={\left[\begin{array}{cc} J_{2} & J_{3} \end{array}\right]}^{-1}\left(\dot{X}-J_{1}{\dot{\theta }}_{1}\right)$$

(48) $$\left[\begin{array}{c} {\ddot{\theta }}_{2}\\ {\ddot{\theta }}_{3} \end{array}\right]={\left[\begin{array}{cc} J_{2} & J_{3} \end{array}\right]}^{-1}\left(\ddot{X}-\dot{J}\left(\theta ,\dot{\theta }\right)-J_{1}{\ddot{\theta }}_{1}\right)$$

### 3.4. Optimal Joint Configuration Based on DP Algorithm

According to Equation (46), when the state of the foot end is unchanged, the amount of control is different, resulting in different motion states of each joint, leading to different energy losses generated. Therefore, this section utilizes the DP algorithm and the energy loss model of Equation (44) to obtain the joint configuration with the lowest energy loss.

**Figure 6:**
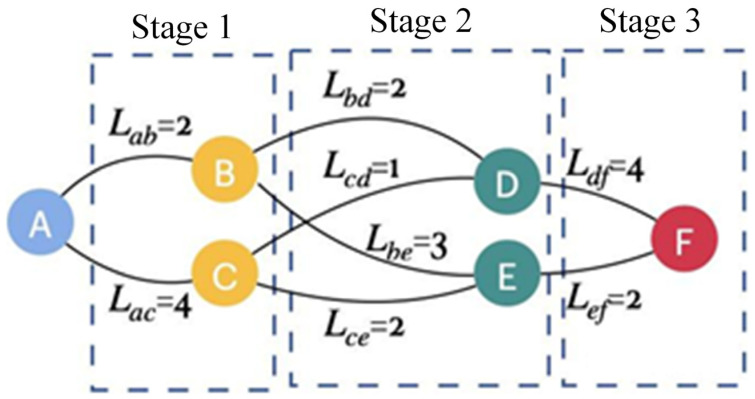
The schematic diagram of DP algorithm.

The DP algorithm is a widely used calculation method for solving continuous decision-making problems with staged decision-making capability, which is able to consider the accumulated cost in decision-making and optimize the total cost over the entire time series, as shown in Figure 6. Therefore, in the energy loss optimization task of the 3-DOF hydraulic single leg in this paper, the cost is the energy loss E during the movement time of the single leg, and the DP algorithm ensures that the energy loss E is minimized by evaluating different $u_{k}$ value sequences. In addition, the method provides not only the final optimization result but also the optimal control action for each time step.

In addition, energy loss $E\left(X,\theta ,u\right)$ is a nonlinear function of time t, foot-end position X, foot-end velocity $\dot{X}$, joint angle θ, and joint angular velocity $\dot{\theta }$. It is worth noting that states X and $\dot{X}$ can be controlled by changing the angular acceleration $\ddot{\theta }$ in inverse kinematics. Assuming that time is discretized into multiple time periods $T_{s}$, $\ddot{\theta }$ is held constant within each $T_{s}$, i.e., $\ddot{\theta }=C$. Thus, for each $T_{s}$, a $\ddot{\theta }$ can be found such that the total energy loss E from the current moment to the final moment is minimized.

Define the cost function as J, which represents the minimum energy loss from the beginning of any state to the end of the final state, and can be expressed as

(49) $$J_{k}\left(\theta_{k}\right)=\underset{u_{k}\in U}{\min}\left\{E_{k}\left(X_{k},\theta_{k},u_{k}\right)+l_{k}\left(X_{k},\theta_{k},u_{k}\right)+J_{k+1}\left(f\left(\theta_{k},u_{k}\right)\right)\right\}$$

where $X_{i}$, $\theta_{i}$, and U are the set of foot-end states $X_{i}\in \{X_{1},X_{2},\ldots ,X_{NX}\}$, the set of joint states $\theta_{i}\in \left\{\theta_{1},\theta_{2},\ldots ,\theta_{N\theta }\right\}$, and the control set U ∈ {$u_{1}$,$u_{2}$,…,$u_{Nu}$}, respectively. $N_{X}$, $N_{\theta }$, and $N_{u}$ are the number of discrete divisions of foot-end state, joint state, and control variables, respectively. $E_{k}\left(X_{k},\theta_{k},u_{k}\right)$ is the energy loss at time k, and $l_{k}$ is the physical constraint penalty term of the joint at time k. The aim is to exclude singular solutions that exceed the limits of joint motion and cause unstable joint motion.

Therefore, for the value range of $N_{X}$, $N_{\theta }$, and $N_{u}$, in order to make the generated data set comprehensive, a total of 125 foot position sampling points are evenly distributed in the foot space; that is, $N_{X}=125$. According to the limit position of each joint motion, a total of 256 joint position configurations are selected on average in the joint configuration; that is, $N_{\theta }=256$. Let the number of control variables $N_{u}=4$. The above data is substituted into the function J, and the data e with the optimal energy loss $E_{\min}\left(X_{i},\theta_{i},u_{i}\right)$ is obtained as the training data of the subsequent feedforward neural network.

The penalty term can be constructed by using the physical limit of the joint, which can be expressed as

(50) $$l=k_{\lim}\cdot \sum_{i=1}^{3}\left\{\begin{array}{l} H\left(\theta_{i}-\theta_{i\max}\right)+H\left({\dot{\theta }}_{i}-{\dot{\theta }}_{i\max}\right)+H\left({\ddot{\theta }}_{i}-{\ddot{\theta }}_{i\max}\right)+\\ H\left(\theta_{i\min}-\theta_{i}\right)+H\left({\dot{\theta }}_{i\min}-{\dot{\theta }}_{i}\right)+H\left({\ddot{\theta }}_{i\min}-{\ddot{\theta }}_{i}\right) \end{array}\right\}$$

where $k_{\lim}$ is the penalty coefficient, $\theta_{i\min}$ and $\theta_{i\max}$ are the joint angular limits due to the physical limits of the HDU, ${\dot{\theta }}_{i\min}$ and ${\dot{\theta }}_{i\max}$ are the joint velocity limits due to the maximum output volume of the hydraulic pump and the cylinder diameter of the HDU, and ${\ddot{\theta }}_{i\min}$ and ${\ddot{\theta }}_{i\max}$ are the joint angular acceleration limits due to the influence of the system pressure, respectively. Define $H\left(x\right)$ as a step function, which can be specifically expressed as

(51) $$H\left(x\right)=\left\{\begin{array}{cc} 1 & x>0\\ 0 & x\leq 0 \end{array}\right.$$

The DP algorithm is used for global motion planning based on energy loss optimization for the 3-DOF hydraulic single leg with the pseudo-code shown in Algorithm 1.

Algorithm 1: The code principle and pseudocode of the DP algorithm are shown below.
**Algorithm 1:** Dynamic programming algorithm  Part 1: Solution in reverse order  Initialize X, θ and $u_{k}$  Calculate energy loss J  for *k* = *N* − *1* to 0  for all $X_{k}\in X$ do          for all $u_{k}$ ∈ U do                    $\theta_{k+1}=f\left(\theta_{k},u_{k}\right)$  Using parametric inverse kinematics to compute θ, $\dot{\theta }$, $\ddot{\theta }$ at time *k*.   Using the joint mapping relationship to calculate $x_{p}$, ${\dot{x}}_{p}$, ${\ddot{x}}_{p}$ at time *k*.  Calculate energy loss J                     $J_{k}\left(\theta_{k}\right)=\underset{u_{k}\in U}{\min}\left\{E_{s,k}\left(X_{k},\theta_{k},u_{k}\right)+l_{k}\left(X_{k},\theta_{k},u_{k}\right)+J_{k+1}\left(f\left(\theta_{k},u_{k}\right)\right)\right\}$         end for                     $J_{k}\left(\theta_{k}\right)=\underset{u_{k}\in U}{\min}{J^{\prime }}_{k+1}\left(x_{k},u_{k}\right)$                     ${u^{\prime }}_{k}\left(\theta_{k}\right)=\underset{u_{k}\in U}{\arg\min}{J^{\prime }}_{k+1}\left(x_{k},u_{k}\right)$         end for end for  Part 2: Forward solution  Read ${u^{\prime }}_{k}\left(\theta_{k}\right)$  for *k* = 0 to *N* − *1* do  Take out ${u^{\prime }}_{k}\left(\theta_{k}\right)$   $\theta_{k+1}=f\left(\theta_{k},u_{k}\right)$  end for                  Using parametric inverse kinematics to compute θ, $\dot{\theta }$, $\ddot{\theta }$ at time *k*.

## 4. Neural Network Learning

In nature, many organisms are constantly evolving to adapt to the changing demands of the environment. And mammals are endowed with an excellent sense of autonomous energy-efficient conservation through natural evolution and learning over time, and are able to fulfill the task requirements with lower energy loss. Therefore, based on the RDOF characteristics of the hydraulic legged robot, an NN is used to learn the inverse kinematics model under minimum energy loss by referring to the autonomous energy-efficient consciousness of mammals.

### 4.1. Structure of Neural Networks

**Figure 7:**
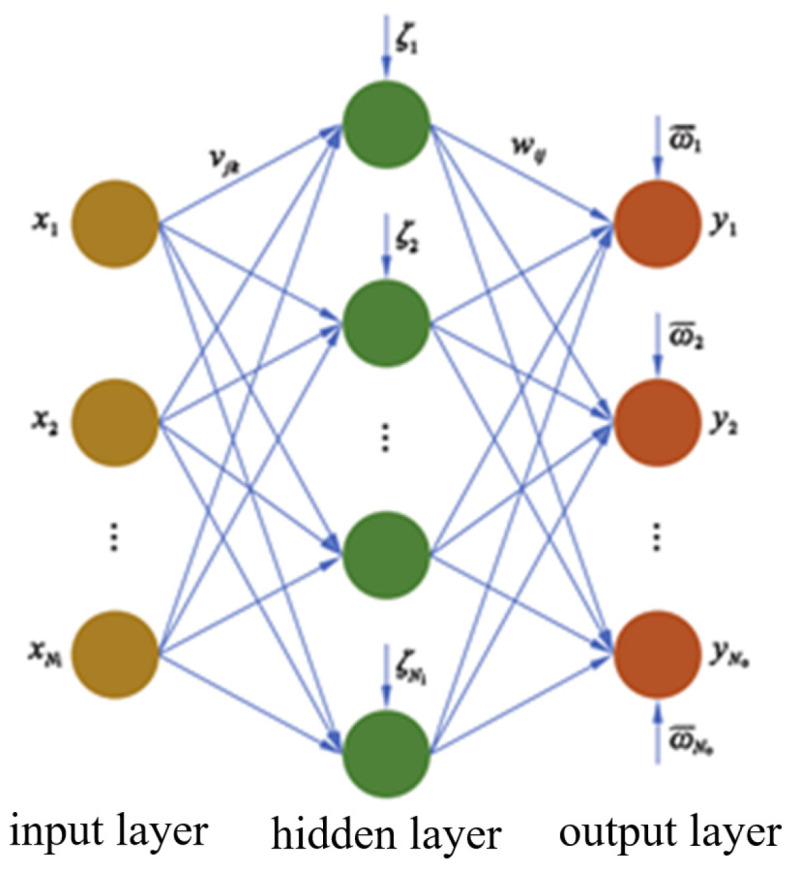
The structure of FNN.

An artificial neural network (ANN) is a mathematical model inspired by biological nerves and used to simulate human brain thinking. A feedforward neural network (FNN) is one of the typical representatives of ANNs that possesses excellent function approximation and parallel processing ability and constructs a high-dimensional complex mapping from input space to output space by superimposing simple nonlinear functions. An FNN is an effective method to deal with the state mapping of complex systems. In this paper, an FNN is used as the basis to establish the energy-efficient inverse kinematics NN model for a 3-DOF hydraulic single leg. As shown in Figure 7, an FNN usually consists of an input layer, a hidden layer, and an output layer. The output of the FNN can be expressed as

(52) $$y_{i}=\sum_{j=1}^{N_{h}}\left(w_{ij}F\left(\sum_{k=1}^{N_{i}}v_{jk}x_{k}+\zeta_{j}\right)+\varpi_{i}\right),i=1,2,\ldots ,N_{o}$$

where $x_{k},y_{i}$ are the inputs and outputs of the NN, respectively, $N_{i},N_{h},N_{o}$ are the number of neurons in the input, hidden, and output layers, respectively, $v_{jk},w_{ij}$ are the weights of the hidden and output layers, respectively, $\zeta_{j},\varpi_{i}$ are the bias terms of the hidden and output layers, respectively, and $F\left(\cdot \right)$ is the activation function of the hidden layer.

**Figure 8:**
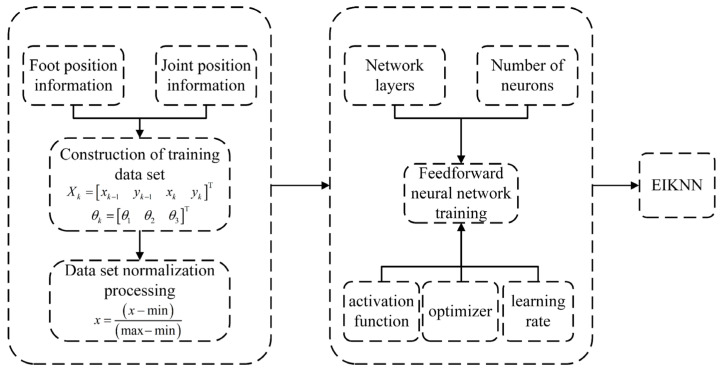
NN structure for energy-efficient inverse kinematics learning.

The advantage of FNNs is that the information transfer between layers is unidirectional, and each neuron in each layer forms a unidirectional connection with all neurons in the next layer without localized closed loops. FNNs have a simple structure, fast calculation speed, and are suitable for large-scale data sets and real-time processing. Therefore, the structure of the NN used for energy-efficient inverse kinematics learning in this paper is shown in Figure 8.

### 4.2. Training Process of Neural Network

**Figure 9:**
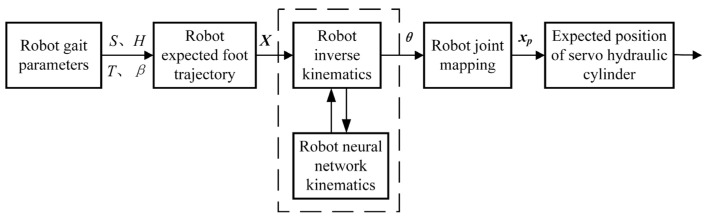
Control principal diagram of EIKNN for hydraulic legged robot.

Based on the data set obtained by the DP algorithm, the NN used for energy-efficient inverse kinematics learning is trained to learn the inverse mapping from the foot-end motion space to the joint configuration space. As shown in Figure 9, during the motion of the hydraulic legged robot, the NN takes the desired foot-end trajectory generated by gait parameters as the input and outputs the joint configuration under the minimum energy loss to completely replace the traditional inverse kinematics calculation process and drive the HDU to complete the motion. Finally, the motion control of the hydraulic legged robot in the energy loss optimization task is completed.

The input of the NN is the foot-end position $X_{k}={\left[\begin{array}{cccc} x_{k-1} & y_{k-1} & x_{k} & y_{k} \end{array}\right]}^{T}$, and the output is the joint angle $\theta_{k}={\left[\begin{array}{ccc} \theta_{1} & \theta_{2} & \theta_{3} \end{array}\right]}^{T}$. In addition, the empirical formula is used to determine the number of nodes in the hidden layer:

(53) $$h=\sqrt{n_{in}+n_{out}}+a$$

where h is the number of nodes in the hidden layer, $n_{in}$ is the number of nodes in the input layer, $n_{out}$ is the number of nodes in the output layer, and a is the experience factor. Its value range is 1–10.

In this paper, because the input feature vector is four-dimensional and the output signal is three-dimensional, the number of hidden layer nodes calculated according to Formula (53) should be between 4 and 13, which is the best. In addition, setting the appropriate learning rate is also crucial for the performance of the neural network. The performance of each iteration will be directly affected by the size of the learning rate: A higher learning rate can make the network quickly approach the optimal solution, but it may lead to increased volatility in the later period, which in turn leads to unstable network performance; although the lower learning rate is stable, it may lead to a training time that is too long and may not achieve convergence within a predetermined number of iterations. The setting range of the general learning rate should be between 0.01 and 0.8. In this paper, the learning rate is set to 0.03, and the maximum number of iterations is 1000.

**Figure 10:**
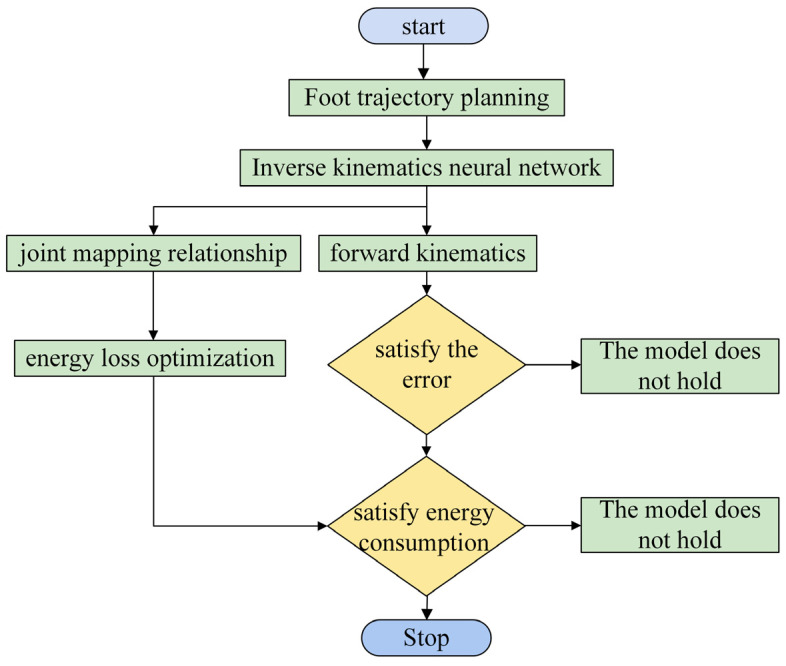
Training process of EIKNN.

According to the training process as shown in Figure 10, the EIKNN model can be obtained.

**Figure 11:**
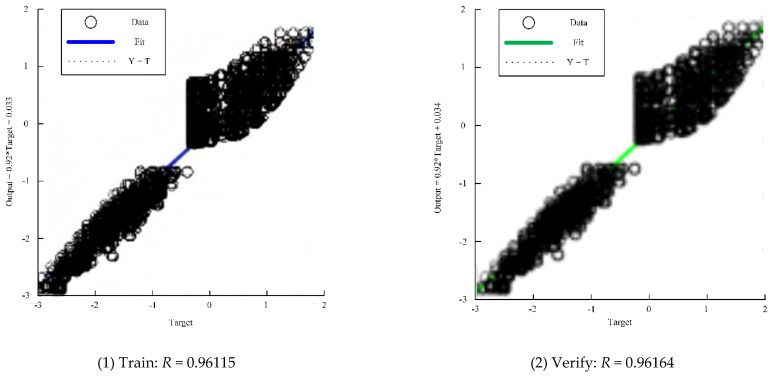

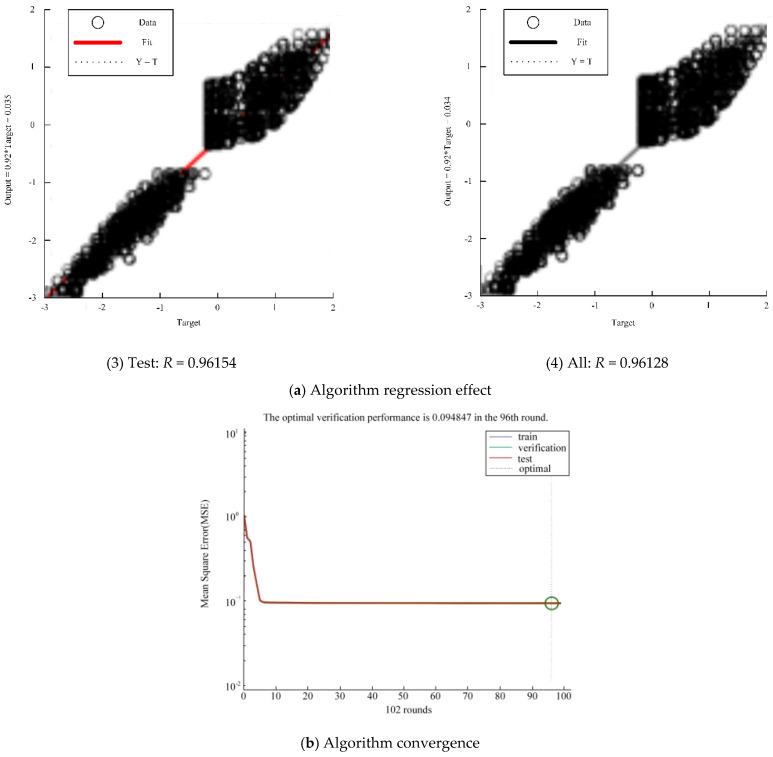
Neural network training results.

The final training results are shown in Table 5 and Figure 11.

## 5. Experimental Verification

### 5.1. Experimental Platform and Scheme Comparison

**Figure 12:**
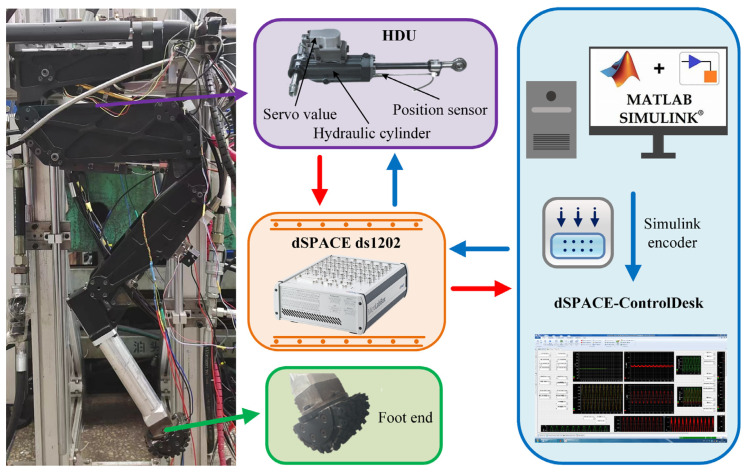
The performance test platform of the 3-DOF hydraulic single leg.

In this section, an experimental validation of the EIKNN will be conducted. As shown in Figure 12, the performance test platform for a 3-DOF hydraulic single leg of a hydraulic legged robot is presented. The physical parameters of the experimental platform are shown in Table 6. The experiment was implemented using MATLAB/Simulink, with the controller employing dSPACE ds1202 at a sampling frequency of 5 Hz.

Here, $K_{xv}$ is the servo valve gain, $C_{ep}$ and $C_{ip}$ are the internal and external leakage coefficients, respectively, $A_{1}$ and $A_{2}$ are the cross-sectional areas of the left and right chambers of the hydraulic cylinder, respectively, $V_{g1}$ and $V_{g2}$ are the volume of the oil inlet pipe and the volume of the oil return pipe, respectively, $p_{s}$ and $p_{0}$ are the system oil supply pressure and the system oil return pressure, respectively, L is the total stroke of the hydraulic cylinder piston, $m_{t}$ is the mass of the hydraulic cylinder piston, $\beta_{e}$ is the effective bulk modulus, K is the load stiffness, and $B_{p}$ is the damping coefficient.

**Figure 13:**
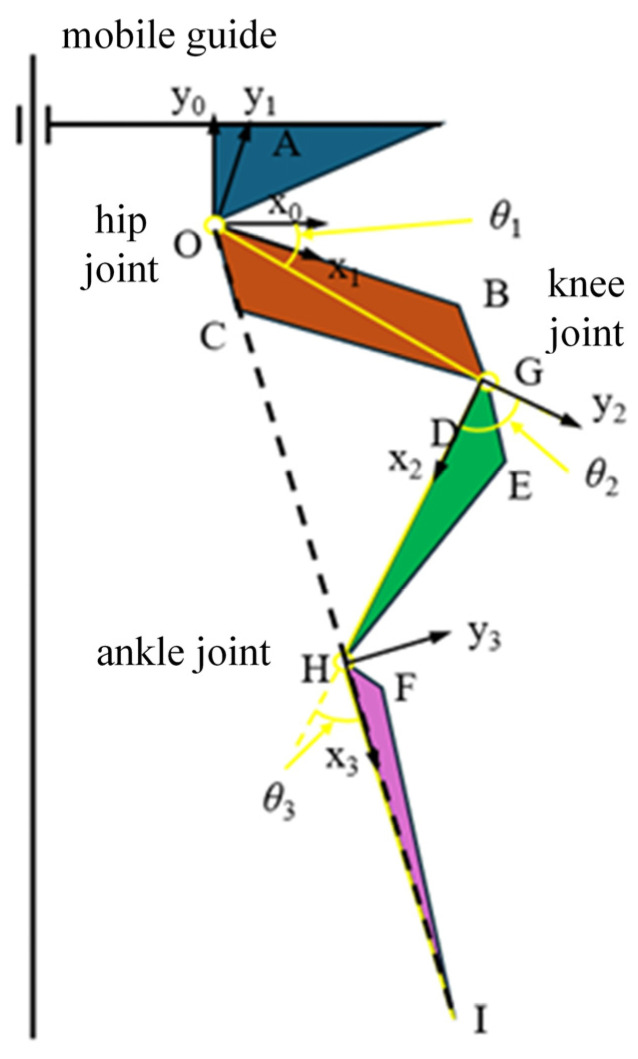
A schematic diagram of the geometric constraints of the robot.

To verify the effectiveness and superiority of the proposed EIKNN, comparative experimental validation was conducted using the following four inverse kinematics solving methods for robots with RDOFs:

(1) This is the EIKNN method proposed in this paper.
(2) Pseudo-inverse matrix method. This method utilizes the pseudo-inverse of the Jacobian matrix to update joint positions. By computing joint velocities ($\dot{\theta }=J^{*}\dot{X}$, where $J^{*}$ is the pseudo-inverse of the Jacobian matrix), it enables rapid joint movements to achieve the desired foot-end velocity. The joint positions are then obtained through time integration until the foot-end position error is minimized. It is worth noting that the pseudo-inverse matrix of the Jacobi matrix does not always exist and may lead to numerical instability when the robot is in a singular configuration. In the comparative approach of this paper, this problem is solved by introducing a regularization term, specifically $\dot{\theta }=J^{T}{\left(JJ^{T}+\lambda^{2}I\right)}^{-1}\dot{X}$.
(3) Gradient projection method. This method searches for joint positions by iterative optimization using gradient information to minimize the error between the actual position of the foot end and the desired position ($e\left(\theta \right)=X_{d}-X\left(\theta \right)$, where $X_{d}$ is the desired position of the foot end, and $X\left(\theta \right)$ is the actual position of the foot end with the joint angle θ as the variable). The iteration step size is set to 0.001. The boundary constraint is set to 0<δ< Trajectory amplitude/100.
(4) Geometric constraint method. This method performs an inverse kinematics solution by adding geometric constraints. In this paper, the 3-DOF hydraulic single leg is added with the motion constraints as shown in Figure 13 to ensure that the three points of the hip joint O, the ankle joint H, and the foot end I are kept in a straight line, thereby obtaining an accurate resolution of the inverse kinematics. However, this method reduces the motion space of the foot end. In addition, the inverse kinematics model of this method is not the focus of this paper; we omit the detailed derivation process and directly give the inverse kinematics expression as

(54) $$\left\{\begin{array}{l} \theta_{1}=\arctan2(y,x)+\arccos\left(\frac{OE^{2}+{\left(\sqrt{x^{2}+y^{2}}-GI\right)}^{2}-EG^{2}}{2\cdot OE\cdot \left(\sqrt{x^{2}+y^{2}}-GI\right)}\right)\\ \theta_{2}=\arccos\left(\frac{OE^{2}+EG^{2}-{\left(\sqrt{x^{2}+y^{2}}-GI\right)}^{2}}{2\cdot OE\cdot EG}\right)-\pi \\ \theta_{3}=\arccos\left(\frac{{\left(\sqrt{x^{2}+y^{2}}-GI\right)}^{2}+EG^{2}-OE^{2}}{2\cdot \left(\sqrt{x^{2}+y^{2}}-GI\right)\cdot EG}\right) \end{array}\right.$$

### 5.2. Experimental Results and Analysis

Case 1: Verification of Solution Accuracy

To validate the effectiveness of the proposed EIKNN method, it is first necessary to verify its solution accuracy. During the experiments, the piston rod displacements of each joint HDU collected by the displacement sensors include both the computational errors of the EIKNN and the control errors of the HDU. Therefore, we used two test methods, including control error and not including control error, for experimental verification.

Test Method 1 (experimental curves include the influence of control errors): Using the desired foot-end trajectory as input to the control system, the expected displacements of each joint HDU piston rod were obtained through EIKNN computation. The actual foot-end trajectory was then collected for comparison. This method yields the foot-end trajectory that incorporates both the computational errors of the EIKNN and the control errors.

Test Method 2 (experimental curves exclude the influence of control errors): The theoretical foot-end trajectory is calculated using a positive kinematics model based on the desired displacement of the HDU piston rod obtained in method 1. This method yields the foot-end trajectory that contains only the computational errors of the EIKNN, while excluding control errors.

**Figure 14:**
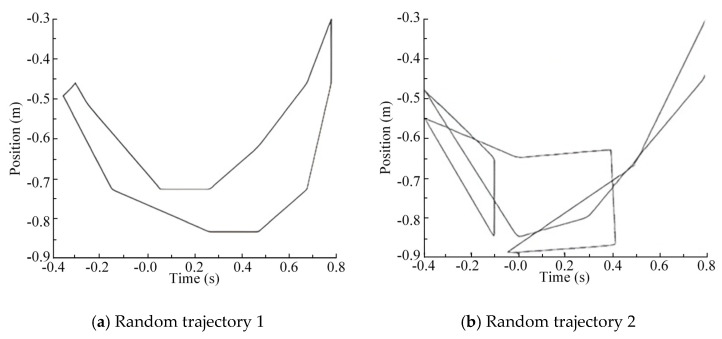
Two sets of random foot-end motion trajectories.

The above two test methods are used to verify the actual performance of the EIKNN by stripping out the effects generated by the control errors. In order to verify the reliability of the proposed EIKNN, random points are taken and connected in the motion space, so that the generated trajectories try to fill the foot-end motion space as much as possible, and the generated random foot-end trajectories are shown in Figure 14.

**Figure 15:**
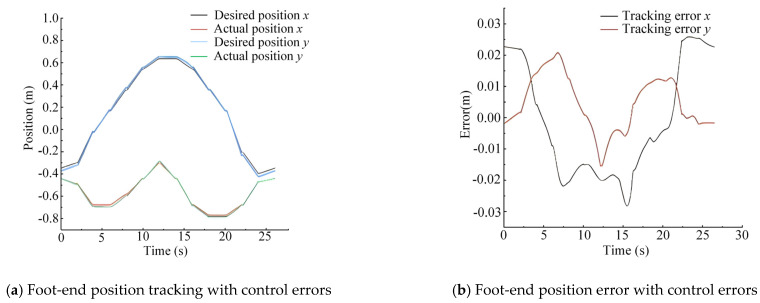

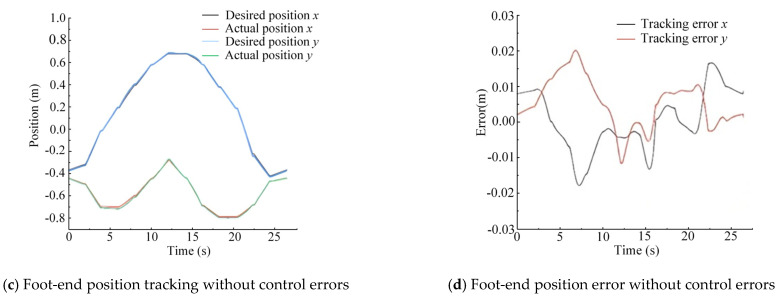
Verification of EIKNN solution accuracy under random trajectory 1.

**Figure 16:**
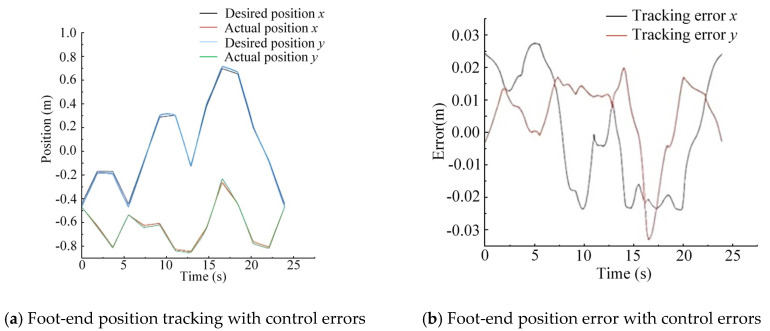

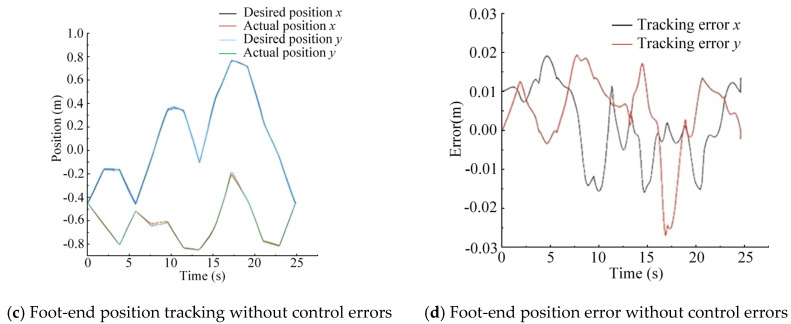
Verification of EIKNN solution accuracy under random trajectory 2.

The two sets of random trajectories in Figure 14 are used as desired foot-end trajectories. As demonstrated by the experimental results in Figure 15 and Figure 16, the foot-end trajectories closely followed the desired trajectories well, confirming the effectiveness of the proposed EIKNN. Furthermore, the joint trajectories generated by the EIKNN exhibit certain positional errors, with the tracking error of the foot end in the y-direction consistently slightly larger than that in the x-direction. Notably, as shown in Table 7, in the two sets of randomized trajectories that included the effect of control error, the peak error of the foot end of the leg as a percentage of the motion travel in that direction was 2.68% and 2.40% in the *x*-axis and 4.40% and 5.00% in the y-direction, respectively. In the two sets of random trajectories that did not include the effect of control error, the peak error of the foot end of the leg as a proportion of the motion travel in that direction was 1.63% and 1.50% in the x-direction and 4.00% and 3.91% in the y-direction, respectively. Consequently, in the x-direction, the errors introduced by the EIKNN are comparable to those caused by control errors, while in the y-direction, EIKNN-generated errors are slightly greater than those attributable to control errors.

Case 2: Energy-saving Verification

**Figure 17:**
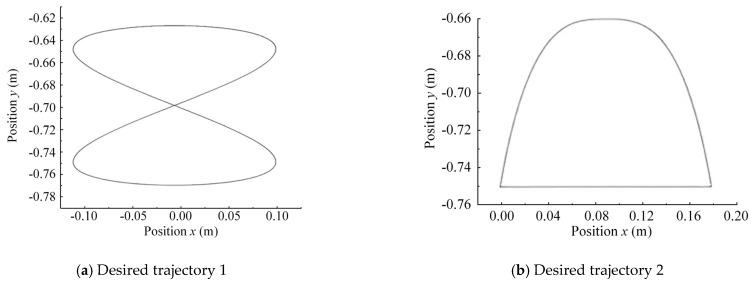
Two sets of desired foot-end trajectories.

To validate the energy-saving performance of EIKNN, two sets of desired foot-end trajectories shown in Figure 17 were given to further test and compare the energy loss between the proposed EIKNN and existing methods under identical trajectory conditions.

**Figure 18:**
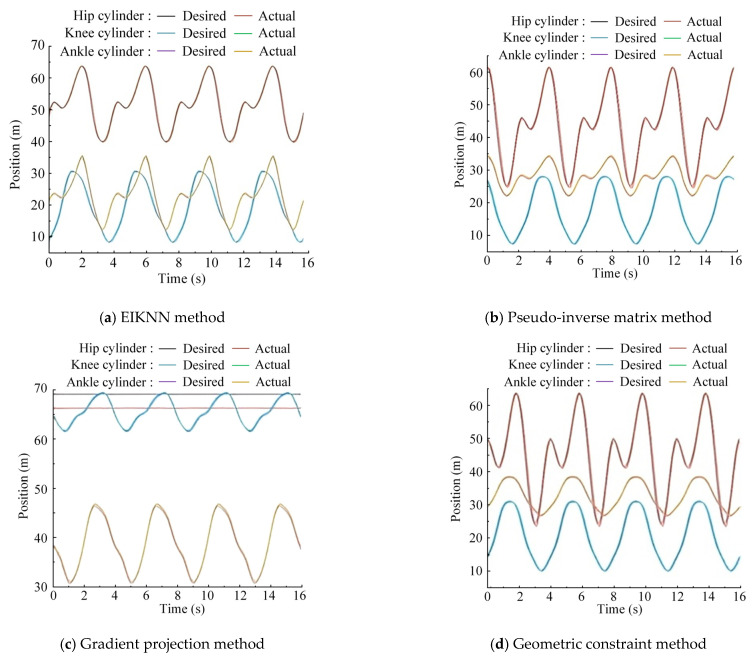
Joint HDU trajectory tracking experimental curves under desired trajectory 1.

**Figure 19:**
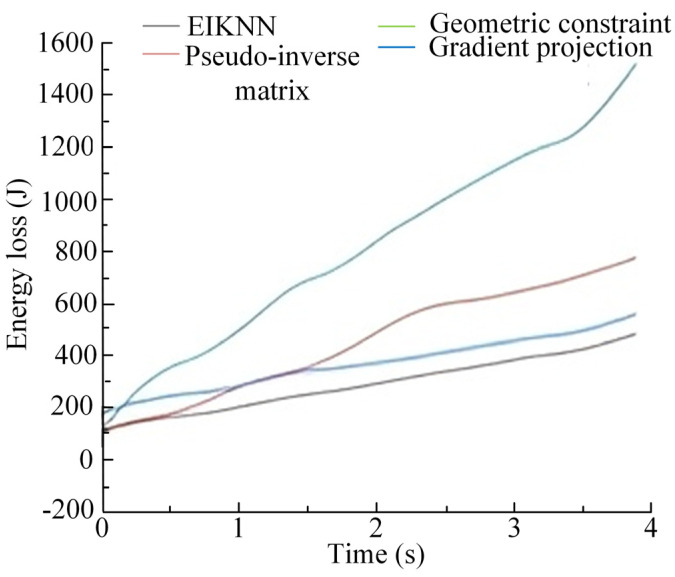
Comparison curves of leg motion energy loss under desired trajectory 1.

**Figure 20:**
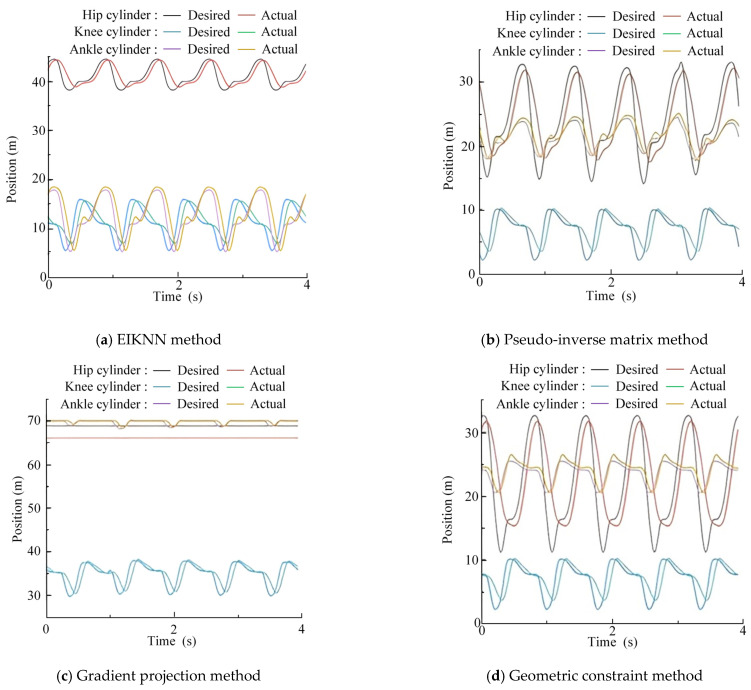
Joint HDU trajectory tracking experimental curves under desired trajectory 2.

**Figure 21:**
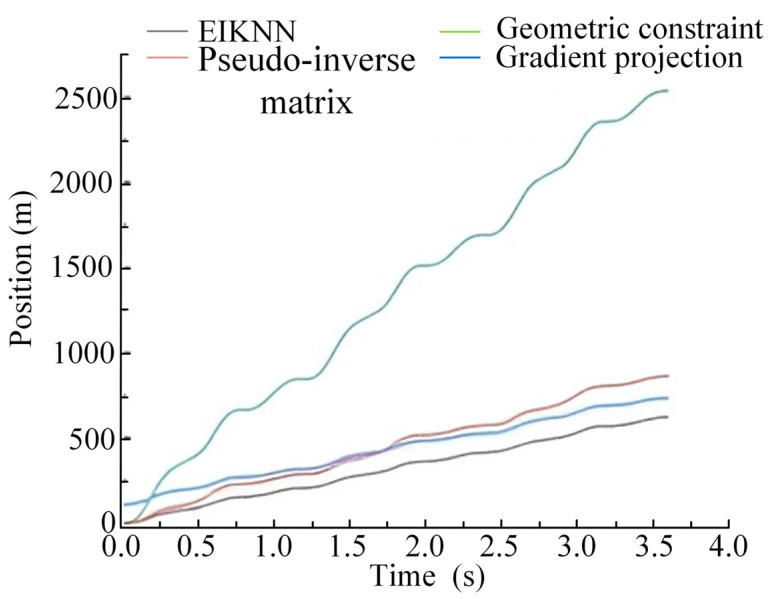
Comparison curves of leg motion energy loss under desired trajectory 2.

The two sets of trajectories in Figure 17 are employed as desired foot-end trajectories, with four distinct inverse kinematics solving methods applied to control the motion of the 3-DOF hydraulic single leg. The experimental results demonstrate that the EIKNN proposed in this paper has good joint HDU trajectory tracking performance and the lowest energy loss. In addition, it can be seen from Figure 18c and Figure 20c that the joint angles tend to exceed the limit position when using the gradient projection method as an inverse kinematics solution under the same kinematic trajectory, resulting in a poorer foot-end trajectory tracking performance after using this method. As illustrated in Figure 20b, when the pseudo-inverse matrix is employed as the inverse kinematics solution method, although the robot does not exceed physical limitations during motion, it exhibits obvious cumulative errors that consequently reduce the stability of leg movement. In contrast, Figure 18a and Figure 20a demonstrate that under different desired trajectories, our proposed EIKNN method maintains stable tracking performance without any observable cumulative error. Furthermore, as evidenced by Figure 19 and Figure 21, the energy loss curve of the EIKNN method shows the smallest slope compared to the other three methods, indicating the minimal energy loss during the operation period. These results conclusively validate the superior energy-saving performance of the proposed EIKNN method.

Case 3: Further verification of energy-saving

To further validate the joint motion performance and energy-saving effects achieved through EIKNN computation, two sets of foot-end trajectories were generated via trajectory interpolation based on the start and end positions specified in Table 8. The geometric constraint method was selected as the control group in this experiment to prevent both joint error accumulation and exceedance of motion limits during single-leg movement.

**Figure 22:**
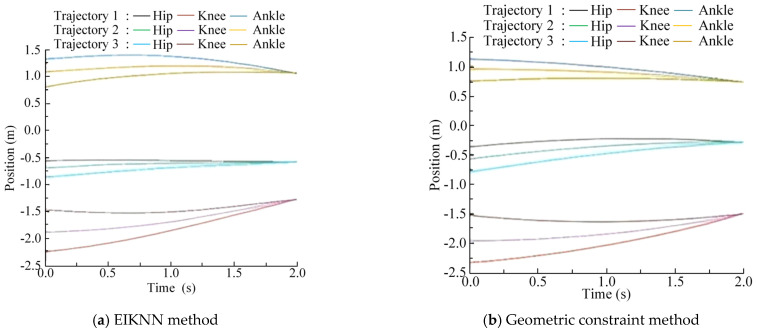
Position of the joints under scheme 1.

**Figure 23:**
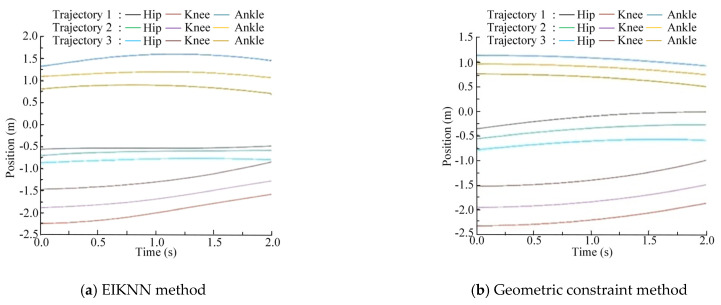
Position of the joints under scheme 2.

**Figure 24:**
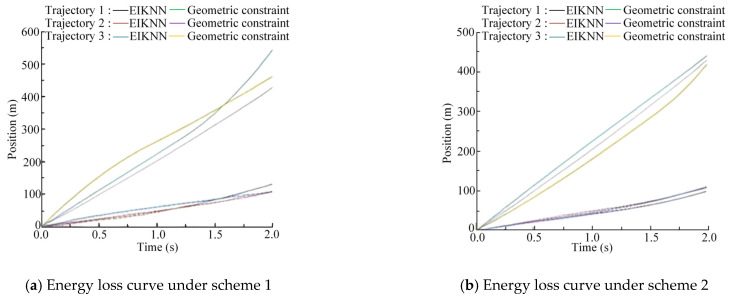
Energy loss under two schemes.

As illustrated in Figure 22, Figure 23 and Figure 24, compared with the geometric constraint method, the EIKNN proposed in this paper has a smaller motion change amplitude in the hip and knee joints after using the EIKNN to solve the joint position under the same foot-end trajectory. In addition, the motion amplitude of the hip joint is significantly smaller than that of the knee and ankle joints. This phenomenon is related to the loads applied to the HDUs at each joint. During the single-leg motion, the hip joint HDU experiences the greatest load, resulting in increased energy loss. Therefore, under the condition of satisfying the robot’s motion requirements, the proposed EIKNN method reduces the motion amplitude of the heavily loaded hip joint HDU, thereby minimizing additional energy loss and achieving improved energy efficiency.

## 6. Conclusions

In this paper, inspired by the excellent autonomous energy-efficient consciousness endowed by mammals through natural evolution for a long time, a novel EIKNN method is proposed for hydraulic legged robots with RDOFs, which realizes the seamless integration of leg motion planning and energy saving for hydraulic legged robots. Firstly, the spatial mapping relationship between the HDU and the joint of the leg of hydraulic legged robots is derived, the foot-end motion space under the joint motion limit is analyzed, and the inverse kinematics model of a hydraulic single leg with RDOFs is established. Subsequently, an energy loss model for evaluating leg motion is established, and the DP algorithm is used to solve the optimal joint configuration to minimize the energy loss during motion, and a data set is built for training. Finally, an NN model for energy-efficient inverse kinematics of the leg of a hydraulic legged robot is trained by using an NN to simulate the excellent autonomous energy-efficient awareness that mammals have been endowed with through natural evolution. The experimental results show that the proposed method not only fully meets the demand for solving accuracy in the leg motion of the hydraulic legged robot but also effectively reduces the energy loss. This method has good adaptability and generalizability for a variety of redundant serial robots and provides a strong and reliable technical support for future research on energy-saving control of high-performance robot systems with RDOFs.

Using DP alone is not feasible for online applications, and using an NN alone cannot guarantee energy-optimal performance. The combination of offline DP for generating optimal labels and an online NN for fast inference ensures both near-optimal power performance and real-time control capability.

In the future, we will focus on a task-specific or structured sampling mechanism in the next work and integrate the motion task and environmental interaction information more systematically into the training data set to enhance the adaptability of the neural network model and the actual control effect. The proposed control strategy is embedded in a more complete dynamic control framework to systematically evaluate the stability and energy efficiency performance of the hydraulic legged robot under more complex environmental conditions.

## Figures and Tables

**Table 1:** Structural parameters of leg components.

Parameter	OA	OB	OG	GC	GD	GH	HE	HF	HI
Value (mm)	85	245	300	249	44	310	248	46	359
Parameter	∠HGD	∠BOG	∠OGC	∠GHE	∠IHF	β	-	-	-
Value (°)	1	12	13.5	13	37.35	65			

**Table 2:** Physical limits of HDUs and joints on leg.

Parameter	Minimum Value	Maximum Value
Position of HDU (m)	−0.07	0.07
Velocity of HDU (m/s)	−0.1	0.1
Acceleration of HDU (m/s^2^)	−0.5	0.5
Rotation angle of hip joint $\theta_{1}$ (°)	−54.439	−4.618
Rotation angle of knee joint $\theta_{2}$ (°)	−137.587	−32.156
Rotation angle of ankle joint $\theta_{3}$ (°)	−4.5412	94.5897

**Table 3:** The resistance coefficient of fluid along the pipeline under different conditions.

Fluid Flow Type	Different Kinds of Pipes	Value of λ
Laminar flow	Circular pipe	λ=75/Re
	Curved pipe	λ=82/Re
		λ=155/Re
	Hose with small bending radius	λ=108/Re
	Straight pipe with standard pipe joint	λ=75~85/Re

**Table 4:** LuGre friction microscopic model.

Parameter	Value
$v_{s}$ (m/s)	0.0103
$F_{c}$ (N)	2.4440
$F_{s}$ (N)	0.5991
$\sigma_{0}$ (Ns/m)	0.4766
$\sigma_{1}$ (Ns/m)	0.2701
$\sigma_{2}$ (Ns/m)	0.0049

**Table 5:** Neural network training process.

Unit	Initial Value	Stop Value	Target Value
Round	0	102	1000
Duration	-	00:00:17	-
Performance	1.05	0.0955	0
Gradient	1.39	0.000169	1 × 10^−7^
Mu	0.001	1 × 10^−6^	1 × 10^10^
Verification Check	0	6	6

**Table 6:** The physical parameters of the test bench for the 3-DOF hydraulic single leg.

Parameter	Value	Unit	Parameter	Value	Unit
$K_{xv}$	4.5 × 10^−4^	m/V	$p_{0}$	0.5 × 10^7^	Pa
$C_{ep}$	0	m^3^/(s * Pa)	$C_{ip}$	2.38 × 10^−13^	m^3^/(s * Pa)
$A_{1}$	5.97 × 10^−4^	m^2^	$m_{t}$	1.1315	kg
$A_{2}$	3.97 × 10^−4^	m^2^	$\beta_{e}$	8.0 × 10^8^	Pa
$V_{g1}$	6.2 × 10^−7^	m^3^	K	0	N/m
$V_{g2}$	8.6 × 10^−7^	m^3^	$B_{p}$	2000	N/(m/s)
L	0.07	m	$p_{s}$	1.0 × 10^7^	Pa

**Table 7:** The effect of foot-end trajectory tracking.

Foot-End Trajectory Tracking		Trajectory Amplitude *x* (m)	Error Amplitude *x* (m)	Error Rate	Trajectory Amplitude *y* (m)	Error Amplitude *y* (m)	Error Rate
Random trajectory 1	Testing method 1	1.1	0.0295	2.68%	0.5	0.022	4.40%
	Testing method 2	1.1	0.018	1.63%	0.5	0.02	4.00%
Random trajectory 2	Testing method 1	1.27	0.0305	2.40%	0.64	0.032	5.00%
	Testing method 2	1.27	0.019	1.50%	0.64	0.025	3.91%

**Table 8:** Parameters for generating foot-end trajectory.

Trajectory Scheme		Starting Point	Ending Point
Scheme 1 (Different starting points with the same ending point)	Trajectory 1	((0, −0.6)	(0.4, −0.7)
	Trajectory 2	(0, −0.7)	(0.4, −0.7)
	Trajectory 3	(0, −0.8)	(0.4, −0.7)
Scheme 2 (Different starting points with different ending points)	Trajectory 1	(0, −0.6)	(0.4, −0.6)
	Trajectory 2	(0, −0.7)	(0.4, −0.7)
	Trajectory 3	(0, −0.8)	(0.4, −0.8)

## Data Availability

The data presented in this study are available in the main text.

## References

[1] Gao J., Jin H., Gao L., Zhu Y., Zhao J., Cai H. (2025). Jump Control Based on Nonlinear Wheel-Spring-Loaded Inverted Pendulum Model: Validation of a Wheeled-Bipedal Robot with Single-Degree-of-Freedom Legs. Biomimetics.

[2] Zhao L., Yu Z., Han L., Chen X., Qiu X., Huang Q. (2023). Compliant motion control of wheel-legged humanoid robot on rough terrains. IEEE/ASME Trans. Mechatron..

[3] Lee S., Yoon S., Jeong Y., Seo J., Park S., Han S., Kim J.T., Kim J., Choi H.R., Cho J. (2023). Design and implementation of a two-wheeled inverted pendulum robot with a sliding mechanism for off-road transportation. IEEE Rob. Autom. Lett..

[4] Kamegawa T., Akiyama T., Sakai S., Fujii K., Une K., Wang Y., Matsumura Y., Kishutani T., Nose E., Yoshizaki Y. (2020). Development of a separable search-and-rescue robot composed of a mobile robot and a snake robot. Adv. Rob..

[5] Zhen T., Yan L. (2023). Real-time control strategy of exoskeleton locomotion trajectory based on multi-modal fusion. J. Bionic Eng..

[6] Tong K., Li M., Qin J., Ma Q., Zhang J., Liu Q. (2024). Differential game-based control for nonlinear human–robot interaction system with unknown desired trajectory. IEEE Trans. Cybern..

[7] Du G., Deng Y., Ng W.W., Li D. (2022). An intelligent interaction framework for teleoperation based on human-machine cooperation. IEEE Trans. Human-Machine Syst..

[8] Zhou Q., Yang S., Jiang X., Zhang D., Chi W., Chen K., Zhang S., Li J., Zhang J., Wang R. (2023). Max: A wheeled-legged quadruped robot for multimodal agile locomotion. IEEE Trans. Autom. Sci. Eng..

[9] Zhang K., Zong H., Zhou L., Zhang J., Fang L., Ai J., Xu B. (2025). Design and parameter identification of model-based control integrating hydraulic cylinders in robotic leg dynamics. Control Eng. Pract..

[10] Cho B., Kim S.W., Shin S., Oh J.H., Park H.S., Park H.W. (2022). Energy-efficient hydraulic pump control for legged robots using model predictive control. IEEE/ASME Trans. Mechatron..

[11] Zong H., Zhang J., Jiang L., Zhang K., Shen J., Lu Z., Wang K., Wang Y., Xu B. (2024). Bionic lightweight design of limb leg units for hydraulic quadruped robots by additive manufacturing and topology optimization. Bio-Des. Manuf..

[12] Zhao J., Zhang Y., Hou H., Yue Y., Meng K., Yang Z. (2024). Active Disturbance Rejection Control With Backstepping for Decoupling Control of Hydraulic Driven Lower Limb Exoskeleton Robot. IEEE Trans. Ind. Electron..

[13] Lee Y.H., Lee Y.H., Lee H., Kang H., Lee J.H., Phan L.T., Jin S., Kim Y.B., Seok D.-Y., Lee S.Y. (2020). Development of a quadruped robot system with torque-controllable modular actuator unit. IEEE Trans. Ind. Electron..

[14] Huang J., An H., Yang Y., Wu C., Wei Q., Ma H. (2020). Model predictive trajectory tracking control of electro-hydraulic actuator in legged robot with multi-scale online estimator. IEEE Access.

[15] Xie Z., Jin L., Luo X., Sun Z., Liu M. (2020). RNN for repetitive motion generation of redundant robot manipulators: An orthogonal projection-based scheme. IEEE Trans. Neural Networks Learn. Syst..

[16] Jin L., Li S., Luo X., Li Y., Qin B. (2018). Neural dynamics for cooperative control of redundant robot manipulators. IEEE Trans. Ind. Inf..

[17] Palmieri G., Scoccia C. (2021). Motion planning and control of redundant manipulators for dynamical obstacle avoidance. Machines.

[18] Wen K., Gosselin C. (2021). Exploiting redundancies for workspace enlargement and joint trajectory optimization of a kinematically redundant hybrid parallel robot. J. Mech. Rob..

[19] Fang D., Shang J., Yang J., Wang Z., Xue Y., Wu W. (2018). An Energy Efficient Two-Stage Supply Pressure Hydraulic System for the Downhole Traction Robot. Math. Probl. Eng..

[20] Shao J., Bian Y., Yang M., Liu G. (2022). Characteristic analysis and motion control of a novel ball double-screw hydraulic robot joint. Eng. Appl. Comput. Fluid Mech..

[21] Li X., Luo L., Zhao H., Ge D., Ding H. (2023). Inverse Kinematics Solution Based on Redundancy Modeling and Desired Behaviors Optimization for Dual Mobile Manipulators. Intell. Robot. Syst..

[22] Ba K.X., Yu B., Kong X.D., Li C.H., Zhu Q.X., Zhao H.L., Kong L.J. (2018). Parameters sensitivity characteristics of highly integrated valve-controlled cylinder force control system. Chin. J. Mech. Eng..

[23] Sulaiman S., Sudheer A.P., Magid E. (2024). Torque control of a wheeled humanoid robot with dual redundant arms. Proc. Inst. Mech. Eng. Part I J. Syst. Control. Eng..

[24] Flight J., Gosselin C. (2024). Kinematically redundant (6+ 2)-dof HEXA robot for singularity avoidance and workspace augmentation. Mech. Mach. Theory.

[25] Deng W., Zhou H., Zhou J., Yao J. (2022). Neural network-based adaptive asymptotic prescribed performance tracking control of hydraulic manipulators. IEEE Trans. Syst. Man Cybern. Syst..

[26] Yang X., Deng W., Yao J. (2022). Neural adaptive dynamic surface asymptotic tracking control of hydraulic manipulators with guaranteed transient performance. IEEE Trans. Neural Networks Learn. Syst..

[27] Kramar V., Kramar O., Kabanov A. (2022). An artificial neural network approach for solving inverse kinematics problem for an anthropomorphic manipulator of robot SAR-401. Machines.

[28] Lu J., Zou T., Jiang X. (2022). A neural network based approach to inverse kinematics problem for general six-axis robots. Sensors.

[29] Sun X. (2020). Kinematics model identification and motion control of robot based on fast learning neural network. J. Ambient Intell. Hum. Comput..

[30] Chen D., Zhang Y. (2017). Robust zeroing neural-dynamics and its time-varying disturbances suppression model applied to mobile robot manipulators. IEEE Trans. Neural Networks Learn. Syst..

[31] Li S., Zhang Y., Jin L. (2016). Kinematic control of redundant manipulators using neural networks. IEEE Trans. Neural Networks Learn. Syst..

[32] Jin L., Li S., La H.M., Luo X. (2017). Manipulability optimization of redundant manipulators using dynamic neural networks. IEEE Trans. Ind. Electron..

[33] Guo D., Xu F., Yan L. (2017). New pseudoinverse-based path-planning scheme with PID characteristic for redundant robot manipulators in the presence of noise. IEEE Trans. Control Syst. Technol..

[34] Boudreau R., Léger J., Tinaou H., Gallant A. (2018). Dynamic analysis and optimization of a kinematically redundant planar parallel manipulator. Trans. Can. Soc. Mech. Eng..

[35] Hoyt D.F., Taylor C.R. (1981). Gait and the energetics of locomotion in horses. Nature.

[36] Hoyt D.F., Wickler S.J., Dutto D.J., Catterfeld G.E., Johnsen D. (2006). What are the relations between mechanics, gait parameters, and energetics in terrestrial locomotion ?. J. Exp. Zool. A Comp. Exp. Biol..

[37] Yang K., Li Y., Zhou L., Rong X. (2019). Energy efficient foot trajectory of trot motion for hydraulic quadruped robot. Energies.

[38] Cui Z., Rong X., Li Y. (2022). Design and control method of a hydraulic power unit for a wheel-legged robot. J. Mech. Sci. Technol..

